# Practitioners’ views on the measurement and management of postural hypotension in general practice: a qualitative inquiry

**DOI:** 10.3399/BJGP.2024.0695

**Published:** 2025-09-23

**Authors:** Rosina Cross, Sinéad TJ McDonagh, Bethan Treadgold, Jane Masoli, Judit Konya, Gary Abel, James P Sheppard, Bethany Jakubowski, Cini Bhanu, Jayne Fordham, Sarah Lamb, Katrina Turner, Rupert A Payne, Richard McManus, John Campbell, Christopher E Clark

**Affiliations:** 1 Exeter Collaboration for Academic Primary Care, Department of Health and Community Sciences, Faculty of Health and Life Sciences, University of Exeter, Exeter, UK; 2 Nuffield Department of Primary Care Health Sciences, University of Oxford, Oxford, UK; 3 King’s Health Partners, King’s College London, London, UK; 4 Primary Care and Population Health, University College London, London, UK; 5 Mid Devon Medical Practice, Tiverton, UK; 6 Population Health Sciences, Bristol Medical School, University of Bristol, Bristol, UK; 7 dean, Brighton and Sussex Medical School, Brighton, UK

**Keywords:** blood pressure, delivery of health care, hypotension, postural, primary health care, qualitative research, time pressure

## Abstract

**Background:**

Postural hypotension is associated with cognitive decline, falls, and all-cause mortality, representing a substantial burden on the NHS. Postural hypotension is often asymptomatic, making detection and treatment difficult. Currently, there is no systematic approach to measuring and managing postural hypotension in UK general practice.

**Aim:**

To explore barriers to and facilitators of improving postural hypotension measurement and management.

**Design and setting:**

This was a qualitative interview study with healthcare practitioners (HCPs) in general practices in England.

**Method:**

Individual, remote, semi-structured interviews were conducted with a range of HCPs who measure blood pressure in general practice to explore their views and experiences of measuring and managing postural hypotension. Participants were identified from expressions of interest during a national survey. Interviews were video- and audio-recorded, transcribed verbatim, and analysed thematically.

**Results:**

In total, 26 HCPs in 24 practices across nine clinical research networks in England were interviewed between March and July 2023. HCPs checked for postural hypotension when patients were older, reported dizziness, fatigue, or had chronic conditions. Despite awareness of clinical guidelines, various diagnostic definitions were provided and measurement protocols varied between participants. Sit-to-stand rather than supine-to-stand measurements were considered more feasible owing to time constraints and patient mobility. Education and training, as well as incentives and specialist clinics, were suggested as methods to improve postural hypotension measurement and management.

**Conclusion:**

This is the first study, to our knowledge, to explore barriers to and facilitators of postural hypotension measurement in English general practice. Findings suggest a more systematic approach to measurement is needed to improve detection and management of postural hypotension in general practice.

## How this fits in

Postural hypotension (PH) is associated with cognitive decline, falls, and all-cause mortality, yet is rarely checked or recorded by healthcare practitioners (HCPs) in general practice across England. This study found that, when HCPs assess PH, a sit-to-stand method is preferred over supine-to-stand, being more feasible given time constraints and patient mobility. Despite awareness of guidelines, HCPs reported varied approaches to measurement, including differences in timing, frequency, and posture. They typically check only older patients, those with symptoms, or with chronic disease. Suggested improvements included education and training of multidisciplinary team members, incentives, and specialist clinics to support better measurement and management in practice.

## Introduction

Postural hypotension is usually defined as the sustained reduction in blood pressure (BP) of ≥20 mmHg systolic BP and/or ≥10 mmHg diastolic BP within 3 min of standing.^1^ It is associated with increased risks of falls, all-cause mortality, and cognitive decline.^2–4^ Falls are the leading cause of disability and death from injury among people aged >75 years and were estimated to cost the NHS >£2.3 billion per year in 2013 (equivalent to £3.1 billion in 2024). Even this may be an underestimate given population trends to older age and increasing multimorbidity.^5,6^

Our recent meta-analysis of pooled international data from 23 cohorts reported a 19% prevalence of postural hypotension among older adults in primary care settings.^7^ However, examination of routine UK general practice records suggests a much lower postural hypotension prevalence of around 1%.^8^ This may reflect underreporting of the condition by patients, as over half are asymptomatic or present with vague symptoms, as well as underdetection by healthcare practitioners (HCPs) and incomplete coding of diagnoses.^8,9^

Our related national survey of multidisciplinary HCPs working in general practices across England has confirmed that postural hypotension is not routinely considered, measured, or managed according to current guidance. If postural hypotension is checked for, it is typically when patients report symptoms, such as dizziness.^10^ However, postural symptoms are not a reliable indicator of postural hypotension, and there is some confusion around measurement methods and diagnostic thresholds; therefore, a simple, systematic, and practical approach to postural hypotension detection is required if it is to be adopted successfully in busy general practice settings.

To gain further understanding of reasons for underrecognition, undertesting, and underrecording of postural hypotension in primary care we undertook this study to explore HCPs’ experiences and views of current postural hypotension measurement and management strategies, to identify how postural hypotension could be more effectively identified and treated in general practice.

## Method

### Research design

Remote (online) semi-structured interviews were conducted with a range of HCPs responsible for the measurement of BP in general practices across England. Participants were recruited via clinical research networks (CRNs) through a national survey of HCPs aiming to understand how postural hypotension measurement and management is organised in general practice. Participants were able to express willingness to take part in a follow-up interview during the survey.^10^ Stratified purposive sampling was used to maximise sample variance across HCP roles, GP practice size, urbanicity, and the sociodemographic characteristics of the practice. Participants were invited to participate and were sent participant recruitment information via the email they provided in the survey. Full eligibility criteria are listed in Box 1.

**Box 1.** Eligibility criteria

	Criteria
**Inclusion**	Practice-based multidisciplinary healthcare professionals involved in the day-to-day measurement of BP in general practice Able to access the internet via any device Able to provide consent
**Exclusion**	Multidisciplinary healthcare professionals not predominantly practice based Not involved in the day-to-day measurement of BP

BP = blood pressure.

### Data collection

To ensure consistency across the interviews, a topic guide was used (Supplementary Information S1). This guide was informed by the related national survey and co-developed with the study management group (including general practice HCPs) alongside patient and public involvement (PPI) advisors before piloting in local participants. Participants provided written informed consent (Supplementary Information S2) before interviews were conducted by two research team members (the first and third author) using Microsoft Teams. Interviews were video- and audio-recorded then transcribed by an external transcription company (Bristol Transcription Service) under a non-disclosure agreement.

### Analysis

Interview data were analysed using thematic analysis facilitated by NVivo (version 12) software.^11^ A predetermined framework (derived from research aims) with three themes (current postural hypotension measurement in general practice, current postural hypotension management in general practice, and suggestions for improving postural hypotension measurement and management) were initially applied to the interview data, while remaining open to inductive themes emerging from the data. Five research team members (the first, second, third, tenth, and senior author) and two PPI advisors analysed interview data, familiarising themselves with transcripts before independent coding by at least two research team members and a PPI advisor. Themes were reviewed and discussed by the research team during coding. After multiple rounds of coding and discussions among the team, the predetermined framework was deemed appropriate for analysis. To ensure credibility and minimise bias, researchers (the first and third authors) paraphrased participant responses during interviews to confirm correct interpretations of data. Transparency was enhanced by establishing an audit trail of coding decisions, and coders having extensive critical discourse about emerging themes. Rigour was improved by using techniques such as individual and team-based reflection on interview notes, themes, and coding, and employing several multidisciplinary coders.^12^

## Results

Semi-structured interviews were conducted with 26 participants (February–August 2023) (Table 1). Mean interview time was 25 min (range 15–42 min).

**Table 1.** Participant characteristics

ID	Health professional role	Age, years	Gender	Time employed at the practice, years	CRN cluster
P001	Doctor	37	Man	8	1
P002	Doctor	51	Man	20	2
P003	Healthcare assistant	57	Woman	20	3
P004	Paramedic	29	Man	1	4
P005	Phlebotomist	20	Woman	0.5	5
P006	Doctor	40	Man	5	6
P007	Doctor	37	Man	8	5
P008	Advanced nurse practitioner	60	Woman	20	4
P009	Doctor	43	Man	1	7
P010	Doctor	62	Man	1	8
P011	Practice nurse	28	Woman	4	9
P012	Doctor	37	Woman	10	1
P013	Nurse prescriber	44	Woman	5	5
P014	Practice nurse	60	Woman	30	8
P015	Advanced nurse practitioner	57	Woman	4	3
P016	Frailty matron^a^	46	Woman	12	5
P017	Advanced nurse practitioner	54	Woman	30	5
P018	Doctor	60	Man	30	7
P019	Practice nurse	52	Woman	23	9
P020	Doctor	37	Man	10	3
P021	Doctor	54	Man	17	5
P022	Practice nurse	44	Woman	3	7
P023	Paramedic practitioner	36	Man	4	5
P024	Doctor	43	Man	15	9
P025	Advanced nurse practitioner	39	Man	9	5
P026	Doctor	37	Man	5	6

^a^Senior nursing role, specialising in care of older adults and patients with multiple long-term conditions who are considered frail or at risk of frailty. CRN = clinical research network.

**Figure 1.**
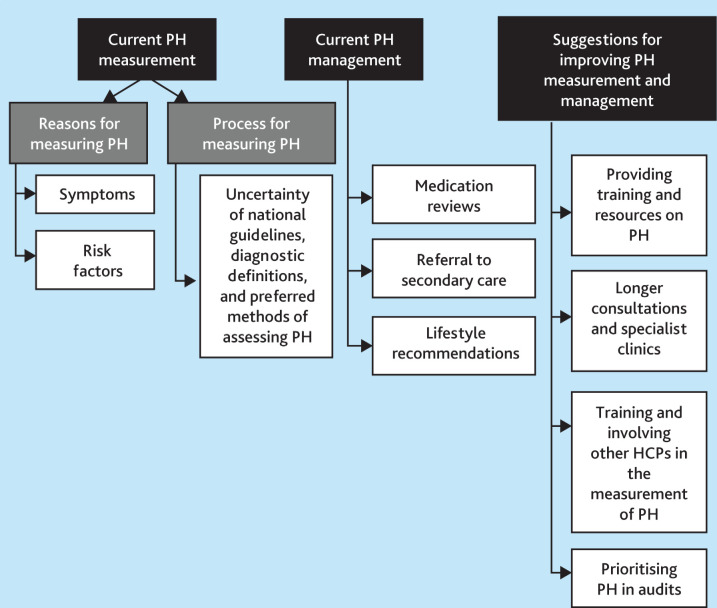
**.** Hierarchy of themes identified in the interview data. HCP = healthcare practitioner. PH = postural hypotension.

After analysis of data coded within the three predetermined themes (current postural hypotension measurement in general practice, current postural hypotension management in general practice, and suggestions for improving postural hypotension measurement and management), we identified 12 sub-themes (Figure 1). Findings are presented under these sub-themes. Where appropriate, participant quotes have been included to illustrate findings.

### Current postural hypotension measurement

#### Reasons for measuring postural hypotension

There were two further sub-themes identified relating to the sub-theme of reasons for measuring postural hypotension: a) symptoms; and b) risk factors.

Relating to the symptoms sub-theme, participants commented that postural hypotension was not typically measured in primary care unless patients report symptoms of the condition:

*‘… it’s not something that’s generally routinely done. So, whilst it probably should be, it isn’t, and it tends to be … when somebody comes for a review, and they mention that they feel dizzy sometimes.’* (P014, practice nurse)

Practitioners reported that, when postural hypotension is measured, it is usually in patients who report dizziness, pre-syncope, fainting, fatigue, or falls history:

*‘I guess I do it from time to time. I would only really do it in someone who has come in with symptoms suggestive of it … I certainly can’t remember doing any routine postural blood pressures*.*’* (P001, doctor)

It was, however, acknowledged by practitioners that problems with BP are often asymptomatic and HCPs need to be cognisant of this:

*‘… just being aware of the fact that blood pressure problems are asymptomatic … They could be experiencing low blood pressure with perhaps not super noticeable symptoms or especially with postural hypotension. People might just think, “Oh it’s normal, like to feel like this.” But actually it’s good for the doctors and nurses to know when they need to check.’* (P005, phlebotomist)

Relating to the risk factors sub-theme, although acknowledging that postural hypotension should be assessed as part of an annual review for those in at-risk groups, such as those on antihypertensive medications, exhibiting frailty, or with chronic conditions such as Parkinson’s disease and/or diabetes, or aged >80 years, many practitioners admitted it was not common practice:

*‘… like those with Parkinson’s who can have other autonomic dysfunction. Do we do that as part of their routine annual reviews? Probably not, actually.*’ (P019, practice nurse)

Furthermore, many practitioners reported uncertainty of the risk factors that were important:

*‘I'm not particularly certain myself. Actually, perhaps older people, elderly people …’* (P005, phlebotomist)

Young adults and students were also identified as an at-risk group by participants who worked in general practices that served predominantly student populations.

#### Process of measuring postural hypotension

A further sub-theme was identified relating to the process of measuring postural hypotension: uncertainty of national guidelines, diagnostic definitions, and preferred methods of assessing postural hypotension. Many practitioners were unaware of postural hypotension guidelines and unsure of the diagnostic definition of postural hypotension:

*‘What are the guidelines? That’s my question! If I’m allowed to know!’* (P024, doctor)

Furthermore, some reported being aware that they or their practice were not following guidelines. For those practitioners who provided a diagnostic definition for postural hypotension, these definitions varied greatly. Some noted a drop in both systolic BP of 20 mmHg and diastolic BP of 10 mmHg, whereas others reported they were only concerned with a drop in systolic BP. Furthermore, one practitioner stated that, if BP values did not meet their diagnostic threshold for postural hypotension, they would use their clinical judgement of symptoms to diagnose presence or absence of postural hypotension:

*‘I just go on more of a clinical judgement and the picture of the patient as to whether they’re symptomatic but no, I don’t actually know what the actual number is*.*’* (P013, nurse prescriber)

Some practitioners reported being aware of National Institute for Health and Care Excellence (NICE) guidelines, and undertaking a lying measure of BP, which is the orthodox method of measurement and detection of postural hypotension:^1^

*‘I suppose I’m a bit pedantic by nature as well so if I want lying/standing, I’m going to try and do lying if at all possible …’ (P024, doctor)*

Those practitioners who were aware of clinical guidelines had credited the following for their awareness: GP updates; being required as a trainer to be ‘on top of’ guideline updates; or having worked in secondary care settings.

Others expressed uncertainty over postural hypotension guidelines. For one GP, this was because they did not consider postural hypotension to be common:

*‘I wouldn’t say I check sitting and standing blood pressure regularly … I very rarely see anyone with serious problems associated with* [postural hypotension], *it means that other factors have to be taken into consideration for the time pressures ... ’* (P002, doctor)

Practitioners conducting a standing BP measurement typically compared this to a sitting rather than a lying measurement and the timings of rest periods before BP measurement varied widely. The commonest reasons for not measuring lying BP were lack of time during appointments, poor mobility of patients (particularly those with complex comorbidity), lack of necessary equipment within consulting rooms (such as a bed), and concerns that the measurement protocol would worsen symptoms:

*‘I think in an ideal world … check their blood pressure lying down as well and getting them to stand from there, but time constraints probably rush me to just do it from sitting and standing.’* (P001, doctor)

In terms of recording postural hypotension diagnoses there was also substantial variation between practitioners: some reported willingness to use clinical codes for postural hypotension but, as a general practice, they believed that they were not coding entries and were more likely to use free-text recordings:

*‘*… *as a practice we’re not very good at doing coded entries. So I think as a practice it would probably be free text …’* (P001, doctor)

This practitioner believed it would be difficult to audit cases. Others agreed but considered that free-text notes made their measurements and diagnoses obvious to colleagues. Clinical coding was reportedly facilitated by the use of templates within clinical systems.

### Current postural hypotension management in primary care

Management of postural hypotension varied according to professional roles and the severity of symptoms. If postural hypotension was suspected based on BP readings, healthcare assistants (HCAs) and nurses generally reported referring patients for review by a GP. GPs themselves reported several different management approaches for postural hypotension, depending on symptom severity.

#### Medication reviews

The commonest management approach by GPs and pharmacists was to review medication, often withdrawing an antihypertensive drug and arranging follow-up BP measurement with a nurse, HCA, or by home or usual ambulatory BP measurement:

*'‘... depends a lot on how bothersome their symptoms are. How dangerous they are. Are they falling? What medications are they on? Is it as a simple as just stopping one of their anti-hypotensive medications or do they need to be considered for referral for testing.’* (P001, doctor)

Medication reviews and alterations were often reported to successfully resolve postural hypotension but, when they did not, practitioners consulted colleagues or referred patients to secondary care for investigation and/or treatment:

*‘I think it varies on the complexity of them ... I can prescribe, I still have a scope of practice where I'd be comfortable or not comfortable with changing things and if I hit my ceiling then I'll seek advice.’* (P023, paramedic practitioner)

#### Referral to secondary care

When patients’ symptoms were not resolved by alterations to medication or patients were increasingly at risk of falls, practitioners referred to secondary care, usually cardiology geriatric departments or falls or frailty assessment clinics. Rarely, patients were referred for a tilt-table test:

*‘… if it’s complex, then I would consider referral to either the falls assessment clinic, or the frailty assessment. So that would be for your complex, frail elderly person who has postural symptoms, who’s on multiple medications and they just need a bit more of a workup.’* (P007, doctor)

#### Lifestyle recommendations

Practitioners reported self-management through lifestyle changes, such as diet (salt intake), staying hydrated, or getting up slowly as alternative approaches that appeared more acceptable to patients than changing medication:

*‘So, I think it’s a lot about how you explain it with the patient and sell the conservative ways of managing it, but overall, like with lots of things, I think people do like to manage these things themselves, rather than changing or adding medications.’* (P007, doctor)

### Suggestions for improving postural hypotension measurement and management

#### Providing training and resources on postural hypotension

Lack of understanding and education on postural hypotension measurement and management were highlighted as issues by many practitioners. Providing postural hypotension training and resources for practice staff was the commonest recommendation to improve postural hypotension measurement and management:

*‘… I think it is something that we do need more education about … I think — the training so that people are actually doing it properly.’ (*P013, nurse prescriber*)*

E-learning modules were the most requested mode of postural hypotension training:

*‘I think nowadays it would probably be online. I know that seems to be everybody’s default sometimes but I do think it is a useful way.’* (P023, paramedic practitioner)

The self-paced nature of online training was reported as preferable:

*‘Yeah, I think for this it would* [be carried out] *online and that it could be done as a recorded webinar so it could be done at any time, and it would involve videos.’* (P024, doctor)

Other training formats mentioned included clinical sessions or bulletin emails within practices, face-to-face training, and production of practice-level postural hypotension protocols.

#### Longer consultations and specialist clinics

Practitioners reported lack of time as the major barrier to appropriate measurement of postural hypotension and believed this could be mitigated through longer consultation times or specialist postural hypotension clinics:

*‘… if we’re doing it properly then we should be doing it properly. Not just sticking it in a 10-minute slot because otherwise you don’t get the reading. You don’t get the correct results, do you?’* (P013, nurse prescriber)

#### Training and involving other HCPs in the measurement of postural hypotension

Practitioners identified HCAs or nurses as being appropriate members of staff to take on the task of measuring postural hypotension if GPs were short of time:

*‘So, I’m confident that they can do that and to be honest with all these sorts of things I always have more confidence in nurses doing them than doctors doing because I think nurses will tend to follow a protocol better and not cut corners as much. That’s my feeling.’* (P024, doctor)

Most practitioners acknowledged that additional training, resources, and support would be needed for HCAs who took up this task. Some practices were already utilising this strategy:

*‘Yeah, and we do have training for our HCAs to do lying and standing blood pressures. So we can book our patients in with them to have that done.*’ (P007, doctor)

#### Prioritising postural hypotension in audits

Many practitioners reported that, if postural hypotension assessment was incentivised or a specific Quality and Outcomes Framework (QOF) target introduced for postural hypotension (a voluntary annual payment-for-performance programme for all general practices in England), there would be greater incentive to measure it. Currently, checking for other auditable conditions, such as hypertension, took priority:

*‘... as GPs we are private contractors and we’re paid essentially per unit of work … but if you wanted to increase the frequency of which it’s done and make it a much more routine test then I think streamlining the process so it’s quicker and paying people for it would be the ways I’d go about it.’* (P009, doctor)

*‘Hm … I think one way … is to somehow have it in QOF ... It could be an indicator for people certainly on blood pressure medication or with diabetes … so those sorts of indicators will often drive activity, so if it was standardly incorporated into reviews for those sorts of conditions.’* (P024, doctor)

Some practitioners suggested, to avoid extra workload associated with an audit, that it could be a task for junior members or registrars to carry out as part of their training:

*‘All the audits that I and my partners do are mostly either CQC* [Care Quality Commission] *audits or prescribing safety audits … but potentially it could be a good one for a trainee to look at …’* (P001, doctor)

## Discussion

### Summary

To our knowledge, this is the first study to explore barriers to and facilitators of postural hypotension measurement and management in general practice in England. Responses suggested a low awareness and understanding of current guidelines for measurement and management of postural hypotension. This included poor understanding of the processes and protocols for measuring postural hypotension in general practice, diagnostic definitions, and identification of people at risk of postural hypotension. When tested for, postural hypotension is usually measured with a sit-to-stand rather than a lying-to-stand protocol. This method is deemed more feasible because of time limitations within consultations, the mobility of patients, and lack of necessary equipment such as a bed. There are also concerns that lying-to-standing measurements could worsen postural symptoms such as dizziness.

This study found that postural hypotension is generally considered for people who report symptoms, rather than for guideline-recommended at-risk groups, such as people with Parkinson’s disease, diabetes, or aged >80 years. Postural hypotension is generally managed in general practice with medication reviews, lifestyle advice, and repeated monitoring; referral to secondary care was considered unusual. Recording and coding of diagnoses of postural hypotension is inconsistent.

Given the lack of understanding surrounding postural hypotension assessment expressed by many practitioners in this study, educational interventions focusing on accurate measurement and management of postural hypotension could be key to improving its detection and treatment. GPs suggested that postural hypotension measurement could be prioritised if it were incentivised through QOF or other targets, making the process a more integral part of routine care.

### Strengths and limitations

The purposive sampling strategy maximised diversity of participants, providing a rich dataset based on varied experiences of multidisciplinary general practice staff. However, it is acknowledged that further sampling of additional participant roles, such as pharmacists and physiotherapists, could have extended our understanding. A common limitation associated with qualitative research is social desirability bias and researcher bias. However, several strategies (paraphrasing participant responses, reflexivity in the approach to data analysis, and having discussions with other researchers) were utilised to increase rigour, credibility, and minimise bias in the study.^12^

### Comparison with existing literature

In line with national survey findings,^10^ we found limited awareness of postural hypotension symptoms or risk markers, suboptimal measurement strategies, uncertainty over diagnostic thresholds, and inconsistent recording and coding of diagnoses. Although the clinical risks of falls, increased all-cause mortality, and greater cognitive decline are recognised, these do not appear to translate into identification of at-risk people to test for postural hypotension.^2–4^

Reference standard consensus assessment for postural hypotension requires a 5-min resting period in a supine position, followed by standing for up to 3 min, both with repeated BP measurements.^1^ This represents a substantial time commitment in busy general practices where workload continues to rise year on year, exacerbated by additional post-COVID-19 pandemic pressures.^13,14^ Therefore, pragmatic shortcuts are adopted; sit-to-standing testing is usually used (and is offered as an initial test in NICE hypotension guidance).^10,15^ These findings, and our previous survey, suggest that adequate rest periods, standing times, and repetition of BP measurements in each position are unlikely to be routinely achieved within currently allocated general practice consultation times.^10^ Specifically, cases of delayed postural hypotension, requiring standing measurements for ≥5 min, will not be detected. Importantly, we have previously shown an association of lower prevalence rates for postural hypotension reported when measurement protocols do not meet the consensus definition (for example, by standing for shorter periods of time) in comparison with protocols that fully implement the consensus. This suggests a risk of further underdiagnosis of postural hypotension if shortened, pragmatic, diagnostic protocols are used.^7^ For this reason, NICE, as of November 2023, recommends formal lying-to-standing assessment if suspected postural hypotension is not confirmed with initial sit-to-stand testing, before refuting the diagnosis.^15^

Internationally, diagnostic criteria vary, risking potential diagnostic uncertainty.^16^ Our participants acknowledged, and were familiar with, NICE guidance, but this did not necessarily lead to implementation, with some interviewees highlighting that they would prioritise clinical judgement and patient presentation over a strict adherence to guidelines in everyday practice.^17^ Previous discrepancies between different NICE guidelines on how to measure postural hypotension were unhelpful in this respect. For this reason, in November 2023, the guidelines for hypotension (NG136) and blackouts (CG109) were harmonised.^15,18^

Interviews highlighted uncertainty and inconsistency in recording and coding diagnoses of postural hypotension; this, in combination with the selection and testing limitations discussed above, may, in part, explain the discrepancy observed between study prevalences (around 20%) and recorded diagnoses (<1%) in routine primary care records of older people.^7,8,19^

### Implications for research and practice

Over 50% of people with documented postural hypotension may be asymptomatic.^9^ HCPs recognise dizziness as a symptom of postural hypotension; however, other symptoms such as coat hanger syndrome, platypnoea, blurry or dimmed vision, or nausea can be vague and underrecognised by both patients and professionals.^9,16,20^ Therefore, a high index of suspicion and better awareness of both symptoms and risk markers is required for HCPs to ensure detection of cases of patients with postural hypotension. In interviews, practitioners highlighted awareness of their lack of understanding as a training need. Various formats for training delivery were suggested, including training modules or webinars, clinical sessions, face-to-face training, or production of practice-level postural hypotension protocols. Online self-paced training content was preferred. Poor awareness of recognised risk markers for postural hypotension was evident, including identification of young students as a particular risk group, in contrast to the evidence that prevalence in fact rises strongly with increasing age and postural hypotension affects <5% of young adults.^3,7^ Education should address these factors, as well as details of formal postural BP measurement protocols, strategies for addressing diagnostic uncertainty, systematic recording of diagnoses, and criteria for considering referral to secondary care.^8^ Further research with key stakeholders (HCPs and patients/carers, and commissioners) is required to develop and implement acceptable and effective training for staff.

Standardisation around an initial sit-to-stand assessment, as suggested in NICE guidance, is pragmatic and in keeping with current practice and resources. However, NICE also recommends subsequent lying-to-standing measurement if clinical suspicion of postural hypotension is not confirmed with initial sit-to-stand testing.^15^ Our findings in this present study, in keeping with the related national survey, suggest that time restrictions are the key limiting factor to achieving this.^10^ Further home and/or ambulatory BP measurement was suggested for follow-up by some interviewees. Such out-of-office measurements, particularly ambulatory nocturnal measurements, can demonstrate episodes of hypotension that may otherwise be missed, or masked in the clinical setting, because of white coat effects, thus permitting additional diagnoses of postural hypotension to be made.^19^

Medication reviews involving discontinuation or reduction in antihypertensive medication were the commonest suggested approach to postural hypotension management. BP trajectories change with time, declining in older age, hence regular review and discontinuation of antihypertensive drugs may become necessary. Postural hypotension can be a trigger for this process, particularly in the context of frailty.^21,22^ Intensification of BP-lowering treatment in response to postural hypotension detection was also raised in interviews. It is the case that the magnitude of postural hypotension can be greater when high BP is uncontrolled,^3,7^ with symptoms improving on intensification of treatment in non-frail older people.^23^ However, this is a complex and poorly understood scenario requiring individual clinical judgement, with minimal high-quality evidence available to guide decision making.^16,24^

Training and education cannot overcome the time and resource implications of adopting per-guideline good-quality postural hypotension testing. Time constraints were identified as a key limitation suggesting that resources need to be allocated to address this, for example, by facilitating longer appointment times for formal postural hypotension testing. Training of HCAs and other team members may free up doctor and/or nurse resources, but interviews also suggested that a strategy to incentivise and remunerate postural hypotension testing could be effective. This could be achieved through inclusion in QOF or other incentive schemes. Although evidence for the longer-term impact of QOFs on outcomes is unclear, remuneration can achieve short-term improvement in testing.^25,26^ Withdrawal of funding, however, is associated with subsequent declines in quality markers so longer-term changes, such as inclusion of an audit cycle as a quality-improvement activity, might also be needed to have an impact on postural hypotension measurement and management in the long term.^27^

In conclusion, there are a range of barriers preventing adequate measurement of postural hypotension. Although those related to lack of awareness may be overcome through education and training, the structural problems of finding adequate time, and sometimes equipment, for accurate postural BP measurement remain. Overcoming these will require allocation of additional resources. Incentivising such testing through remuneration via the QOF or other interventions would be one means of resource allocation and could lead to improved diagnosis, management, and outcomes for people with postural hypotension.
